# Comparing offline decoding performance in physiologically defined neuronal classes

**DOI:** 10.1088/1741-2560/13/2/026004

**Published:** 2016-01-29

**Authors:** Matthew D Best, Kazutaka Takahashi, Aaron J Suminski, Christian Ethier, Lee E Miller, Nicholas G Hatsopoulos

**Affiliations:** 1 Committee on Computational Neuroscience, University of Chicago, Chicago, IL 60637, USA; 2 Department of Organismal Biology and Anatomy, University of Chicago, Chicago, IL 60637, USA; 3 Department of Electrical Engineering and Computer Science, Milwaukee School of Engineering, Milwaukee, WI 53202, USA; 4 Department of Physiology, Northwestern University, Chicago, IL 60601, USA

**Keywords:** motor cortex, spike width, offline decoding

## Abstract

**Objective:**

Recently, several studies have documented the presence of a bimodal distribution of spike waveform widths in primary motor cortex. Although narrow and wide spiking neurons, corresponding to the two modes of the distribution, exhibit different response properties, it remains unknown if these differences give rise to differential decoding performance between these two classes of cells.

**Approach:**

We used a Gaussian mixture model to classify neurons into narrow and wide physiological classes. Using similar-size, random samples of neurons from these two physiological classes, we trained offline decoding models to predict a variety of movement features. We compared offline decoding performance between these two physiologically defined populations of cells.

**Main results:**

We found that narrow spiking neural ensembles decode motor parameters better than wide spiking neural ensembles including kinematics, kinetics, and muscle activity.

**Significance:**

These findings suggest that the utility of neural ensembles in brain machine interfaces may be predicted from their spike waveform widths.

## Introduction

Neural interface systems for control have recently made a number of important advances in recording capabilities, decoding algorithms, and output devices [1, 2]. In particular, these systems have seen nearly a doubling of simultaneously recorded neurons every seven years using either high density electrode arrays, or more recently, optical calcium fluorescence imaging [3]. These advances have provided an ever growing set of rich, high-dimensional signals for control [4]. And yet, decoding ability has not increased correspondingly with the growth of input signals, but rather has plateaued. This disparity arises, in part, because high-dimensional input signals require larger models to relate neural activity to motor features. These models are harder to train for a given size of data, are more prone to overfitting, and thereby less generalizable [5–7]. As such, there is some debate about the optimal size of decoding models [8].

One approach to reduce the dimensionality of neural feature space is to take advantage of the fact that the state space of neural activity patterns is much smaller than the full dimensionality of the neural features being analyzed [9–11]. That is, the responses of individual neurons are correlated, and the number of latent dimensions needed to explain the variability in the ensemble activity is less than the total number of recorded neurons. Indeed, recent reports have shown that the activity of moderately large neural ensembles (tens to hundreds of cells) can be described by a few orthogonal latent dimensions in neural state space [10]. Within this framework, these latent dimensions may be used as control axes for a prosthetic device [11, 12].

Another approach to constrain the dimensionality of neural feature space is to perform model selection using some statistical selection criterion [7]. The guiding principle of this approach is to identify relevant (i.e. predictive) neural features by fitting a model on training data. Several different criteria have been proposed to rank feature relevancy including correlation coefficient [13, 14], mutual information [15], and decoding accuracy [16, 17], while additional criteria, such as Akaike information criterion (AIC) [18], Bayesian information criterion [6, 19], and automatic relevance determination [5, 20] are used to determine the optimal number of features to include in a model.

Instead of approaching model selection as a dimensionality reduction or statistical problem, we wondered if physiological properties of motor cortical neurons could guide the choice of which neurons to use in a decoder. One physiological property that has received much attention recently is spike waveform width. In particular, primary motor cortex (MI) exhibits a bimodal distribution of extracellular, spike waveform widths [21–23]. Although extracellular recordings cannot directly determine cell type or morphology, these two modes exhibit different physiological and functional properties [24]. That study observed that population responses among narrow spiking neurons exhibited more pronounced oscillatory power in the beta frequency band (15–35 Hz) as was observed in the local field potential. Moreover, during peri-movement epochs, narrow, unlike wide spiking neurons, spatially coordinate their firing activity in a way that is consistent with both the wave propagation observed in the local field potential oscillations and the underlying anatomical horizontal connectivity [24, 25]. We reasoned that the narrow spiking neural population may be more closely related to movement because narrow spiking neurons form a network of functional connections that is aligned with the spatial pattern associated with movement onset. Thus, we hypothesized that narrow spiking neurons would lead to more accurate movement decoding as compared to wide spiking neurons.

## Material and methods

### Neurophysiology

All surgical and experimental procedures were approved by either the University of Chicago, or Northwestern University Animal Care and Use Committees, and conformed to the principles outlined in the Guide for the Care and Use of Laboratory Animals (NIH publication no 86–23, revised 1985). Five rhesus macaques (*macaca mulatta*) were implanted with 96 channel Utah electrode arrays in the upper limb area of MI contralateral to their working arm (for details about the exact placement of the electrode arrays, see [26, 27]). Neural signals were collected from these arrays using a Cerebus neural data acquisition system (Blackrock Microsystems, Salt Lake City, UT). Unit spiking activity was sorted offline using semi-manual spike sorting software (Offline Sorter, Plexon Inc., Dallas, TX).

### Behavioral tasks

This experiment consisted of three different tasks involving movement of the upper limb. In the first task, two rhesus macaques were trained to play an instructed-delay, center-out reaching task (for a description of the task see [27]). Briefly, animals were trained to control the position of a cursor using a two-link robotic exoskeleton (BKIN Technologies, Kingston, Ontario, CA). The position of the cursor was projected directly above the position of the animal's hand. A trial began when the animal moved the cursor to a center target and maintained it there for 500 ms. After that time, the animal was cued to move to one of eight possible peripheral targets positioned radially around the center target, then had to wait 1000 ms until a go cue appeared. At this point, the animal was free to move from the center target to the peripheral one. Upon hitting the peripheral target, the animal had to hold the cursor at the peripheral target for 500 ms to complete the trial successfully. Fluid reinforcement was delivered on each successful trial.

Two additional rhesus macaques were trained to play a random target pursuit (RTP) task in multiple experimental conditions (for a detailed description of the task and experimental design see [26]). Briefly, animals used the same robotic exoskeleton to make planar reaching movements to square targets randomly distributed within a 10 × 6 cm workspace. Every time the cursor hit the target, a new target appeared at a random location. In order to complete a trial successfully, an animal had to sequentially hit seven targets. Failure to hit a target within 5 s of its appearance resulted in an aborted trial. Fluid reinforcement was delivered for every successful trial.

One rhesus macaque was trained to perform an isometric wrist flexion task. The upper arm was constrained largely to a para-sagittal plane with the elbow at a 90° angle and the forearm horizontal, in an orientation midway between supinated and pronated. The monkey's wrist was maintained in line with its forearm by securing its hand in a box, which was custom-fit with padding to minimize movement. A six-degree of freedom torque cell was mounted on the box, such that the axes of measurement aligned with those of the wrist. Cursor movement was proportional to the force along the flexion-extension and radial-ulnar deviation axes. The task required the monkey to move the cursor from a central target to one of eight peripheral targets separated by 45°. The force targets were set for each monkey to be submaximal (approximately 20%–30% MVC) in order to reduce fatigue. To initiate a trial, the monkey held the cursor in the central target (requiring no force) for 0.5 s, after which a randomly selected outer target appeared. The monkey was required to move the cursor to the outer target within 5 s, and to maintain that force for 0.5 s in order to receive fluid reinforcement.

### Classification of narrow and wide spiking neurons

We classified units into two discrete physiological classes based on their spike waveform width. To determine the width of each sorted unit, we measured the difference in time between the peak and trough of the average waveform. An additional quantity, waveform signal-to-noise ratio (SNR), was defined as the magnitude of the peak minus the trough in the average waveform divided by the average standard deviation of the waveform across time [26]. Only units with SNRs greater than 3 were used in the subsequent analyses.

For each dataset, a Gaussian mixture model [28] was used to classify spike waveforms into narrow and wide categories. Mathematically, the Gaussian mixture model attempts to describe the distribution of spike waveform widths as a sum of *K* Gaussian distributions. Each Gaussian in the mixture model is referred to as a component (indexed with the variable, *k*), and is fit with a unique mean and standard deviation, *μ_k_*, and *σ_k_*, respectively. Each component also has an additional parameter, *π_k_*, representing the proportion of data described by that component. Expressed as an equation, this model may be specified as

$$p(w)=\sum_{k=1}^{k}\pi_{k}N(w|\mu_{k},\sigma_{k}),$$

where *p*(*w*) is the probability of observing a spike waveform width, *w*, and *N* (*w|μ_k_*, *σ_k_*) indicates a Gaussian distribution with mean, *μ_k_*, and standard deviation, *σ_k_*. This model included an additional regularization parameter, *λ*, that was added to each *σ_k_* to ensure that *σ_k_* remained strictly positive for every component (see [28] for a more complete treatment on fitting Gaussian mixture models. Matlab function fitgmdist, The Mathworks, Natick, MA).

To confirm that the spike waveform width distributions were bimodal, we varied the number of components, *K*, in the mixture model, and computed the AIC, a goodness of fit statistic for each model [29]. As we increased *K*, we also increased *λ* proportionately to ensure that each additional component was non-degenerate. A chi-square test of homogeneity was used to compare the proportion of narrow and wide neurons across recording sessions in a given animal [30].

### Computing other response properties of cells

In addition to determining the waveform width of each cell, we also measured its average firing rate, and, for center-out datasets, the preferred direction and tuning strength. Average firing rate was determined by dividing the spike counts of each cell by the duration of the recording. Firing rate variance was computed using the following formula:

$$\mathrm{var}:=\frac{1}{n-1}\sum_{b=1}^{n}{\left(y_{b}-\bar{y}\right)}^{2},$$

where *n* is the number of 50 ms bins, *y_b_* is the spike count in bin *b*, and *ȳ* is the average spike count over all bins. To determine preferred direction and tuning strength, we fit a cosine-tuning model of the form:

$$y_{i}=\alpha +\beta \cos(x_{i}-\phi )+∊,$$

where *y_i_* indicates the number of spikes between the go cue and target hit on trial *i*, *α* is the overall firing rate of the cell, *β* is the gain of the cosine tuning model, *x_i_* is the angular location of the peripheral target on trial *i*, *ϕ* is the preferred direction of the cell, and *∊* is a normally distributed error term. This model was fit using the Matlab function lsqcurvefit. The tuning strength of the cell was defined as the proportion of variance in spike counts explained by this tuning model.

### Decoding analysis

#### Input features

Spiking activity from every neuron was binned into 50 ms bins. Only neurons with firing rates >1 Hz and waveform SNR > 3 were used in subsequent analyses. The number of neurons that satisfied these criteria is listed in table 1. In general, the spike counts of each neuron in the preceding 20 time bins (i.e. 20 filter taps, 1 s of history) were used as input features to the decoding model, however, we varied the number of taps between 4 and 32 in one analysis to explore the effect of the number of taps on decoding performance (figure 4). In total, the input dimensionality to the decoding model was equal to the number of neurons multiplied by the number of taps (which was 20, unless otherwise noted).

#### Output features

Several different motor related quantities were decoded including kinematic and kinetic features as well as muscle activity. Output features were decoded in 50 ms bins.

In center-out datasets, we decoded shoulder and elbow (joint) torque (computed as described in [31]), joint angular velocities, Cartesian *x* and *y* velocities of the cursor, and wrist speed. In the isometric wrist dataset, j141203, we decoded the activity of 11 muscles of the forearm and hand including extensor digitorum communis (EDC), adductor pollicis longus (APL), flexor digitorum profundis (FDP), extensor carpi radialis (ECR), EDC 2 (EDC2), brachioradialis (Brad), pronator teres (PT), flexor carpi ulnaris (FCU), flexor digitorum superficialis (FDS), flexor carpi radialis (FCR), and FDS 2 (FDS2).

#### Decoding model

All computations were carried out offline in the Matlab programming environment. We employed a standard causal Wiener filter model to decode movement related quantities from neural activity [8, 19, 32–34]. Mathematically, this model satisfies the following objective:

$$\underset{\beta }{\mathrm{argmin}}\sum_{t=1}^{T}\|y_{t}-\alpha -\sum_{u=0}^{19}x_{t-u}\beta_{u}{\|}_{2}^{2},$$

where *y_t_* is a motor quantity at time bin *t*, *x_t–u_* is a vector of spike counts corresponding to time bin *t*—*u*, *α* is an intercept term, *β_u_* is a vector containing the coefficient of each filter tap at time lag, *u*, and ‖ · ‖2 denotes the ℓ_2_ norm. This model is arguably one of the simplest neural decoding models, yet it has been widely used and has been shown to achieve a high degree of decoding accuracy [8, 19, 32]. Model goodness of fit was quantified using the coefficient of determination, *R*^2^ given by the following formula:

$$R^{2}:=1-\frac{\sum_{t}{\left(y_{t}-{\hat{y}}_{t}\right)}^{2}}{\sum_{t}{\left(y_{t}-\bar{y}\right)}^{2}},$$

where *y_t_*, *ŷ_t_*, and *ȳ* denote the observed motor quantity, the fitted motor quantity, and the time averaged motor quantity, respectively.

Models were trained on 75% of available data and tested on the remaining 25%. We found that the proportion of data allocated to training and test sets did not have an appreciable effect on subsequent analyses.

#### Bootstrap analysis

In order to compare decoding performance between narrow and wide spiking neural populations, we drew random ensembles of *N* neurons from each population repeatedly (100 times, with replacement), trained linear decoding models, and measured the decoding performance on a separate set of test data. This process was applied to each dataset individually. The number of neurons in the ensemble, *N*, was varied systematically to quantify how decoding performance scaled in each population.

#### Matching procedure

In order to control for underlying differences in response properties between narrow and wide spiking neural populations, we developed a greedy matching algorithm to select neurons with similar response properties. We chose an ensemble of *N* wide spiking neurons completely at random. Then, for each selected wide spiking neuron, we found the narrow spiking neuron whose response property (e.g. firing rate) was closest to the wide spiking neuron, where closeness was defined by a distance metric (described below for each feature). If the narrow spiking neuron that was closest to the current wide spiking cell was already matched to another wide spiking cell, the next closest unmatched narrow spiking neuron was matched to that wide spiking neuron. We matched several underlying response properties including firing rate, waveform SNR, preferred direction, and tuning strength. For firing rate, waveform SNR, and tuning strength, we used the absolute value of the difference as our distance metric (i.e. the Euclidean distance). For a circular variable like preferred direction, we used the absolute value of the angle between preferred directions as our distance metric.

## Results

**Figure 1:**
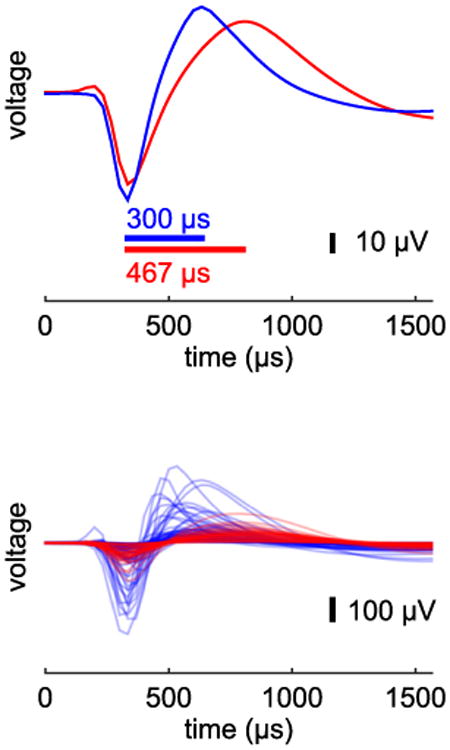
Quantifying spike waveform width. For each sorted unit, we computed its spike waveform width. Here, waveform width is defined as the difference in time between the peak and trough of the average waveform. Exemplary narrow (blue) and wide (red) waveforms (averaged over spikes) are shown as well as the time from trough to peak (top). The distribution of all recorded waveforms from dataset rs050225 (bottom). Color indicates either narrow or wide waveform width.

We recorded spiking activity from single units in primary MI while monkeys engaged in a variety of tasks involving the upper limb. We computed the spike width of each sorted unit (figure 1) and classified it as either narrow or wide. We then compared offline decoding performance of these two classes of cells across many different tasks. A preliminary version of these results was presented as a conference proceeding [35].

### Narrow and wide spiking neural ensembles

**Figure 2:**
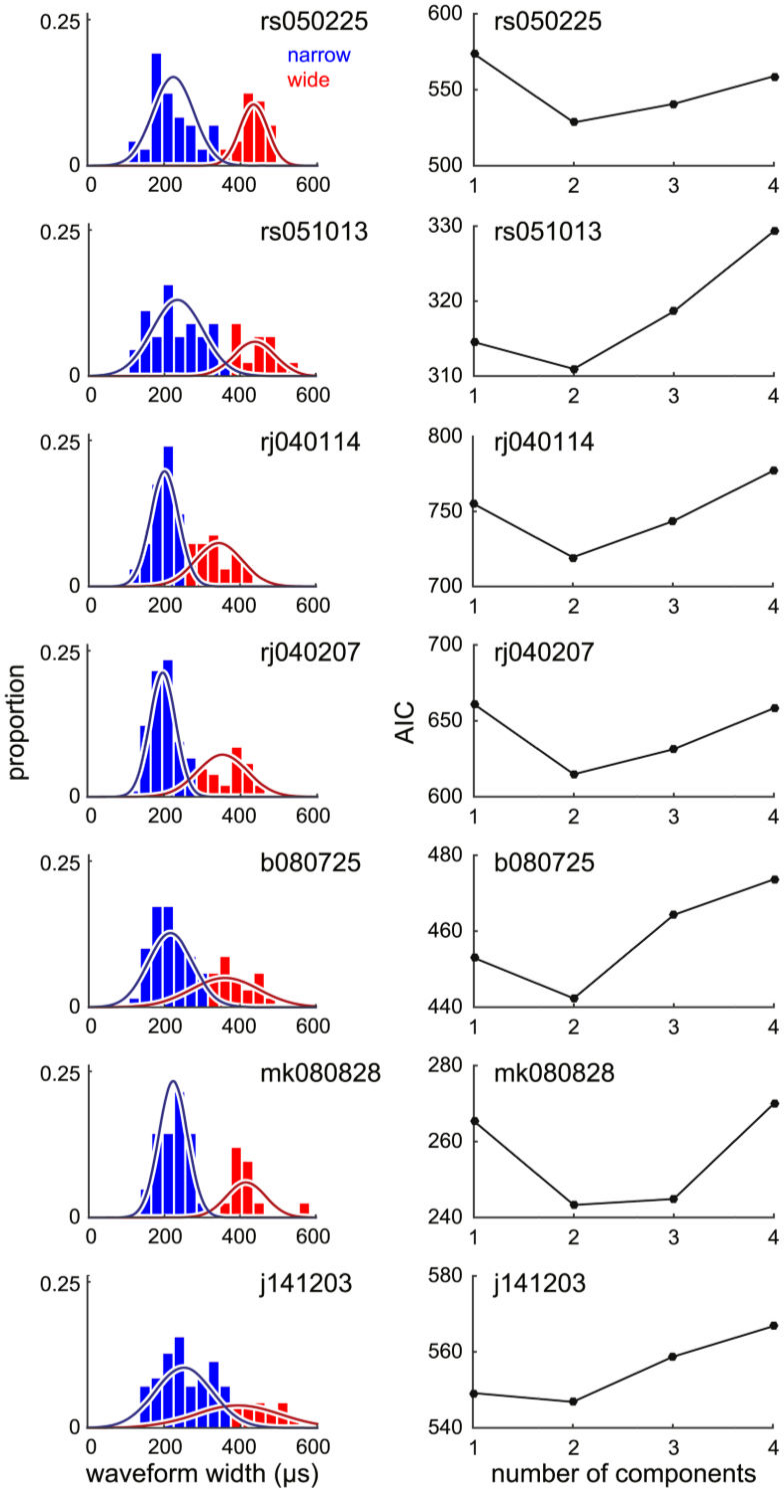
Bimodal distribution of spike waveform widths. A Gaussian mixture model was used to partition neurons from each dataset into narrow and wide spiking categories based on waveform width. To verify that each waveform distribution was indeed bimodal, we systematically varied the number of Gaussians in the mixture model and computed the AIC to perform model selection. For each dataset, we found that a mixture model containing two components best described the data.

We used a Gaussian mixture model to classify neurons as either narrow or wide spiking based on their spike waveform widths (figure 2). A separate model was trained on each recorded dataset (summary statistics of each model given in table 1). To verify that each distribution was indeed bimodal, we fit additional Gaussian mixture models with varied numbers of Gaussian components. For all datasets, we found that a two-component model (i.e. a bimodal distribution) had optimal AIC values. The mean spatial locations of narrow and wide spiking neurons across the cortical sheet were not significantly different (Bonferroni corrected Hotelling's *T*^2^ test) suggesting that subsequent decoding results are not due to differences in the location of the neurons on the cortical sheet.

We examined the consistency of the bimodal distribution across time. In monkeys Rs and Rj, we analyzed datasets that were collected 230 and 24 d apart, respectively. We performed a Chi-square test of homogeneity to assess whether the proportion of narrow spiking units was the same across datasets. We found no evidence of a significant difference in the proportion of narrow spiking units (*p* < 0.42 and *p* < 0.18 for animals Rs and Rj, respectively) across time.

The previous statistical test ensured that the relative proportion of narrow and wide spiking units was the same across time; however, we did not directly gauge whether the average waveform width of each population was similar across time. Accordingly, we performed a *t*-test on the average waveform width of each class across time. In both animals, we found no evidence to suggest that the average waveform width of the narrow spiking class was significantly different across time (Rs: *t*_49_ = −0.82, *p* < 0.21, Rj: *t*_182_ = −0.18, *p* < 0.43). With respect to wide spiking neurons, animal Rs showed no significant difference across time (*t*_20_ = −0.26, *p* < 0.40), although there was a significant difference in Rj (*t*_93_ = − 1.97, *p* < 0.026).

### Decoding kinetics and kinematics

**Figure 3:**
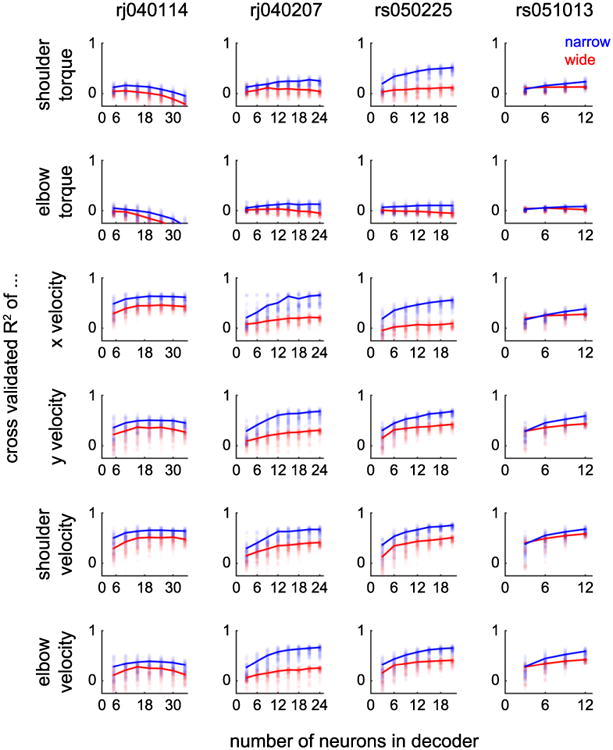
Decoding performance using narrow and wide spiking neural ensembles. We used a standard 20-tap causal Wiener filter to decode kinematic and kinetic quantities from neural data while two animals performed an instructed-delay, center-out task. We repeatedly (100 times) drew random samples of either narrow or wide spiking neurons, trained a decoding model, and then tested its performance on a separate set of data. We found that narrow spiking neural ensembles significantly outperformed wide spiking neural ensembles in a variety of coordinate frames (see text for summary statistics). Each column indicates a different dataset. Individual points correspond to each of the 100 random samples, while the solid lines indicate the upper 75th percentile of decoding performance.

We built simple linear decoding models to predict a variety of kinematic and kinetic motor features based on the activity of either narrow or wide spiking neural ensembles. For our initial analysis, we considered neural data that were collected while animals were performing an instructed-delay, center-out reaching task. We found that narrow spiking neural ensembles outperformed wide spiking neural ensembles at a variety of different ensemble sizes (figure 3, see methods for details about model training and validation). We performed a two-way ANOVA using waveform class (i.e. narrow or wide) and ensemble size as factors. We observed a highly significant main effect of waveform class on decoding performance for each motor feature (median improvement in *R*^2^ was 0.15 across datasets/motor features; *p* < 1e-8 for every dataset/motor feature combination, Bonferroni adjusted for multiple comparisons).

**Figure 4:**
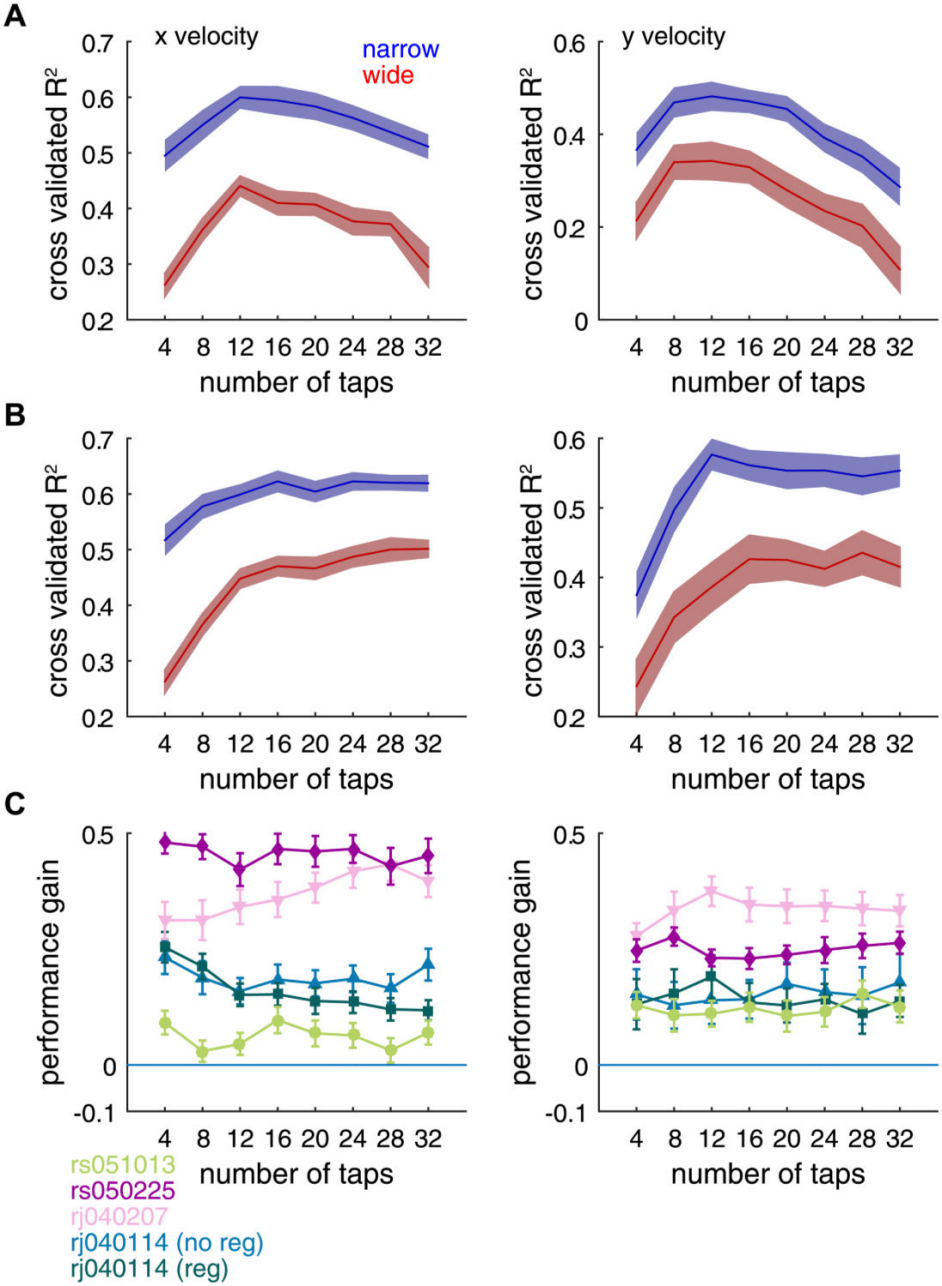
The number of taps does not explain the difference in decoding performance. (A) We fit a linear decoding model containing 20 narrow or wide spiking neurons and systematically varied the number of filter taps. We observed that narrow spiking neurons could predict *x* and *y* velocities (left and right columns, respectively) better than wide spiking populations irrespective of the number of taps. Data shown are from one dataset, rj040114. Solid line indicates average performance across iterations of the bootstrap. Shaded area indicates ± 2 standard errors of the mean. Note that overfitting occurs when using many taps. (B) To ensure that any performance gains were not due to overfitting, we repeated the previous analysis using ridge regression [26]. We observed that decoding performance no longer declined with many taps suggesting that overfitting had been ameliorated by regularization, and that narrow spiking neurons still outperformed wide spiking neurons. (C) We measured the performance gain, defined as the difference in *R*^2^ values between narrow and wide for all datasets and found that the number of filter taps did not explain the disparity in decoding performance. Note that *x* velocity performance gains were slightly larger in the unregularized data from rj040114 suggesting that at least some of the improvement in performance at large numbers of taps may have been due to wide spiking neurons being more overfit than narrow spiking neurons. Nevertheless, in every case, narrow spiking neurons still significantly outperformed wide spiking neurons.

One potential explanation for the difference in decoding performance across waveform classes is that the optimal number of taps for each waveform class could differ. To test this possibility, we fixed the number of neurons in the decoder and systematically varied the number of filter taps from four (200 ms of history) up to 32 (1600 ms of history). Again we observed that narrow spiking neurons outperformed wide spiking neurons irrespective of the number of taps in the model (figure 4(A), ANOVA, *F*_1,784_ = 139.81, *p* < 1e-8, *F*_1,784_ = 407.77, *p* < 1e-8, for *x* and *y* velocity, respectively), or model regularization (figure 4(B), ANOVA, *F*_1,784_ = 628.90, *p* < 1e-8, *F*_1,784_ = 270.81, *p* < 1e-8, for *x* and *y* velocity, respectively). Although decoding performance varied with the number of taps in the model, the relative improvement from using narrow spiking ensembles was fairly constant across the range of taps with the narrow spiking populations always outperforming wide spiking populations (figure 4(C)).

**Figure 5:**
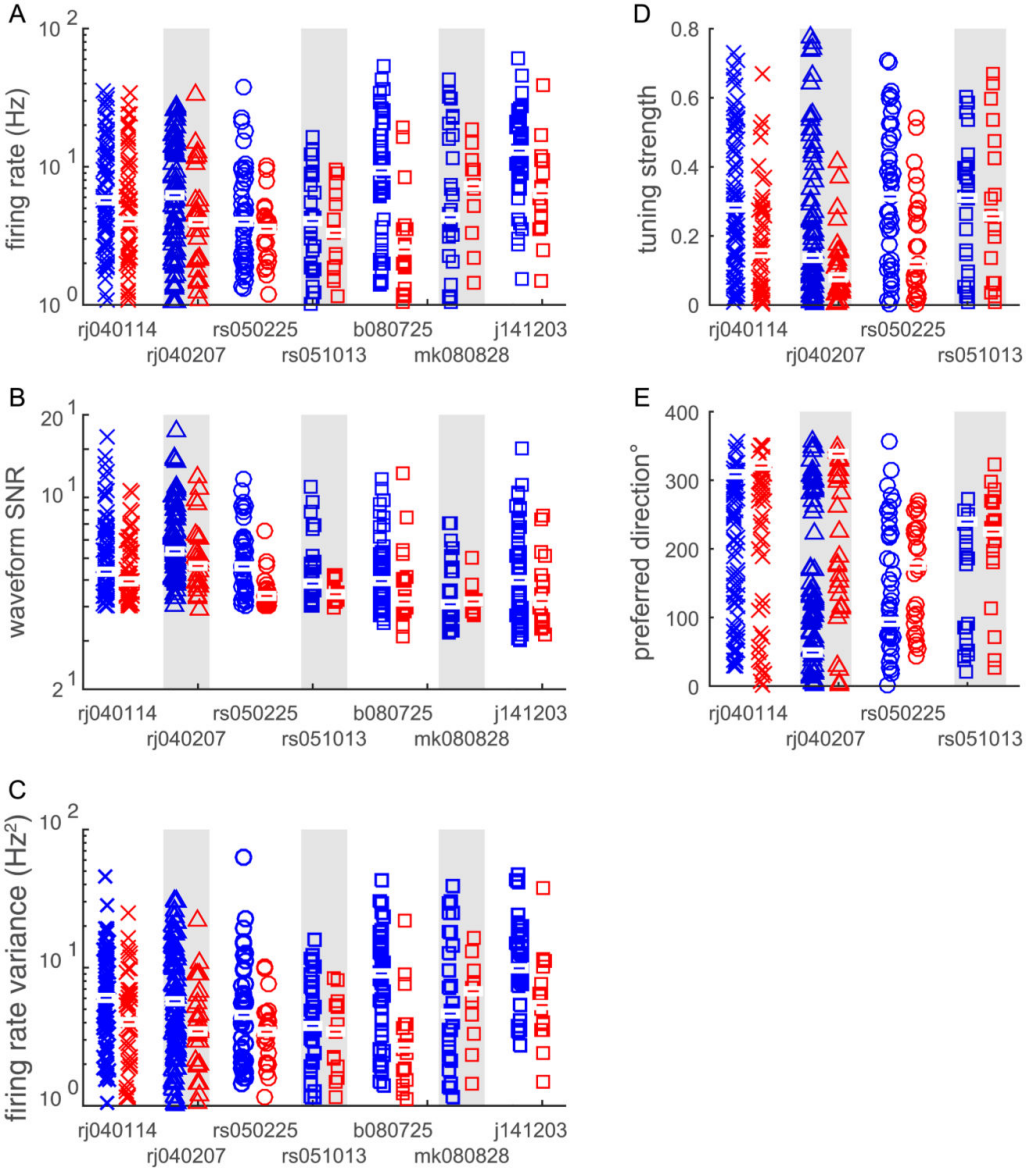
Response properties of narrow and wide spiking neural ensembles. The firing rate, firing variance, and waveform SNR for every neuron from each dataset was estimated (see Methods for details) and then compared based on waveform width category. Generally, narrow spiking units had significantly higher firing rates and waveform SNRs (all datasets except mk080828). Blue and red bars indicate median values for each dataset. For the center-out datasets, we estimated each neuron's tuning strength, and preferred direction. Again, tuning strength was significantly higher for narrow spiking neurons.

Given that the number of taps in the decoder could not explain the difference in decoding ability, we next sought to control for several underlying response properties of these two populations. In general, narrow spiking neurons had higher firing rates, higher firing rate variance, and higher waveform SNRs (figures 5(A)–(C)), although in one dataset, mk080828, wide spiking neurons had higher firing rates and firing rate variance. For two datasets, b080725 and j141203, narrow spiking neuron rates were significantly higher (KS test, *p* < 0.0002 and *p* < 0.003, respectively, Bonferroni adjusted for multiple comparisons), and, narrow spiking neuron rate variability was significantly higher (KS test, *p* < 0.00 002, and *p* < 0.00 006, respectively). Narrow spiking neuron waveform SNRs were significantly greater than wide spiking SNRs in two datasets, rs050225, and rs051013 (KS test, *p* < 0.0002 for both datasets). Additionally, narrow spiking neurons showed stronger directional selectivity as revealed by their higher tuning strengths (figure 5(D)). This trend was significant in both datasets from animal Rj (KS test, *p* < 0.007 for both datasets). However, there was no significant difference in the distribution of preferred directions across waveform class in any dataset (circular medians test [36], *p* > 0.05 for all datasets, figure 5(E)).

**Figure 6:**
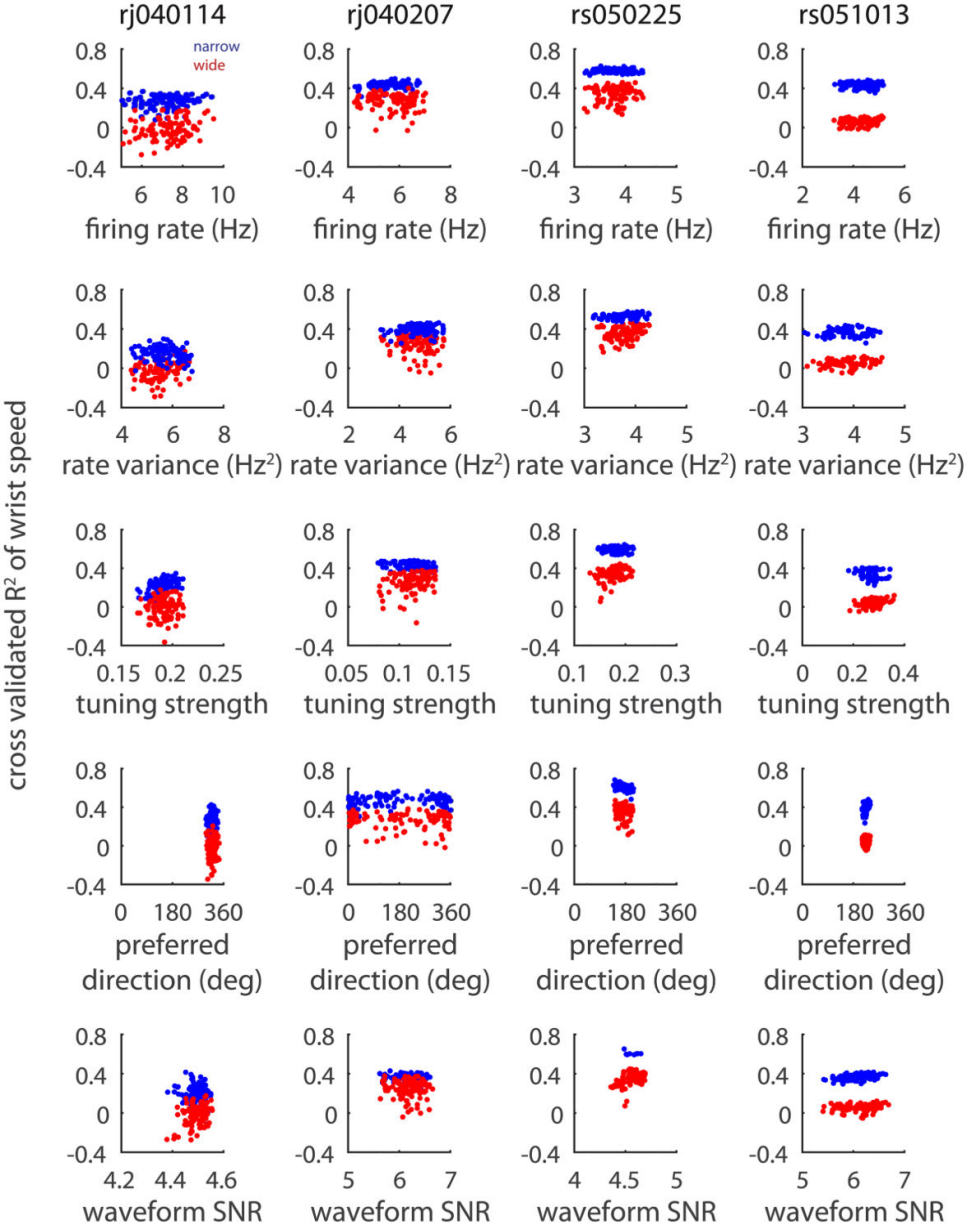
Narrow spiking neurons outperform wide spiking populations even after controlling for differences in response properties. We repeatedly drew random samples of narrow and wide spiking neurons (ensemble size of 30 for rj040114, 20 for rj040207 and rs050225, and 10 for rs051013) while controlling for either firing rate, firing variance, tuning strength, preferred direction, or waveform SNR using a matching procedure (see Methods for details). Narrow spiking neurons outperformed wide spiking neurons even after controlling for differences in response properties.

**Figure 7:**
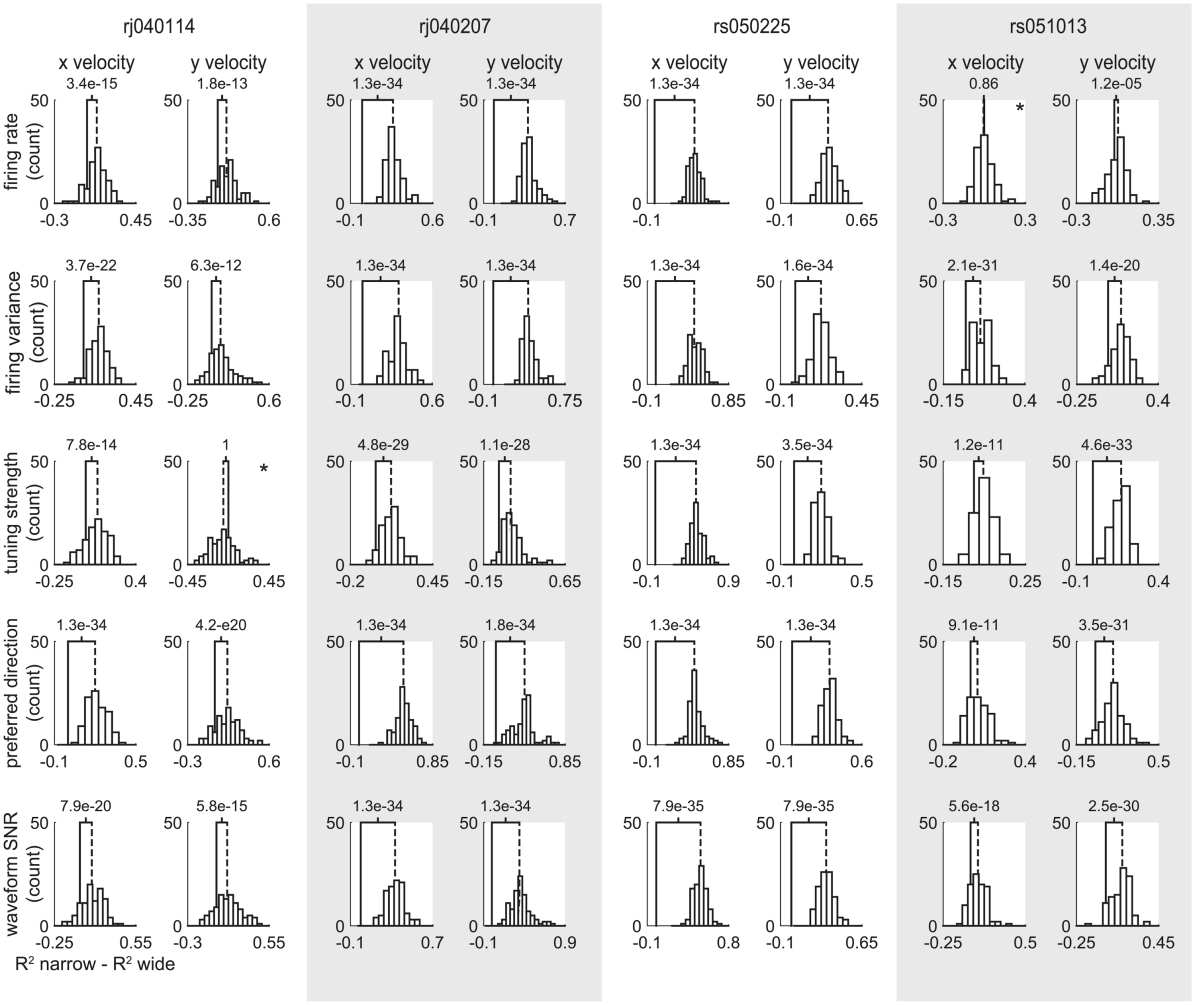
Underlying differences in response properties do not explain the difference in decoding performance. Here we show histograms of the difference in decoding performance (of *x* and *y* velocity) between narrow and wide spiking populations. The average difference is indicated by a vertical dashed line, while 0 is indicated by the solid vertical line. In almost every instance (except two indicated by stars), narrow spiking units outperformed wide spiking units even after controlling for one response property.

We developed a matching procedure to control for any putative differences between narrow and wide spiking neurons (see Methods for details). Random samples of wide spiking neurons were matched with narrow spiking units that exhibited the same firing rate, waveform SNR, preferred direction, or tuning strength each independently. This matching procedure yielded samples of narrow and wide spiking neurons that had statistically indistinguishable averages. Even after controlling for one underlying response property, narrow spiking units still almost always outperformed wide spiking units across a variety of motor features (figure 6 for wrist speed and figure 7 for *x* and *y*, velocities).

### Decoding muscle activity

**Figure 8:**
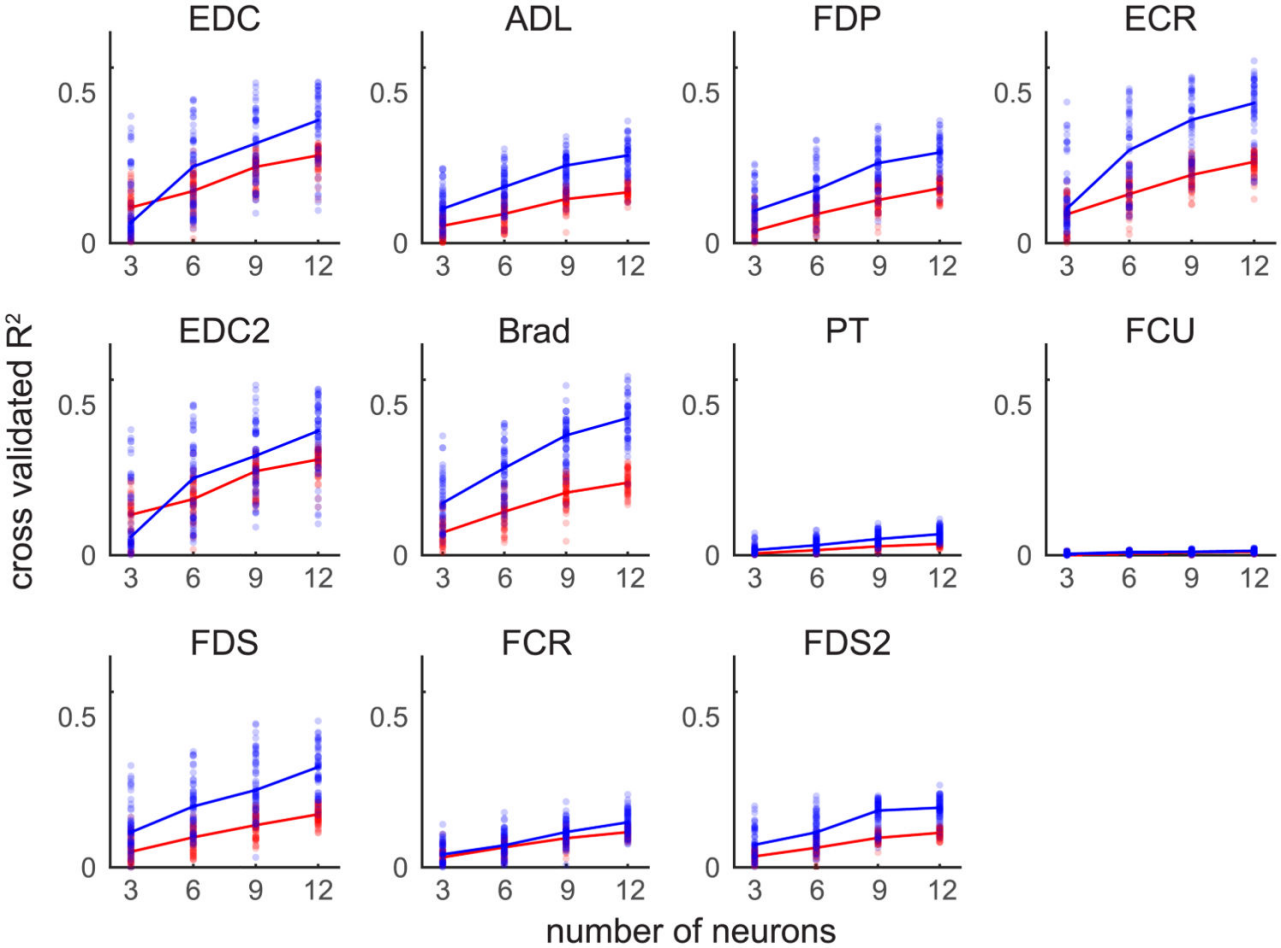
Decoding muscle activity using narrow and wide neural ensembles We used a standard 20-tap causal Wiener filter to decode muscle activity from neural data while an animal performed an isometric wrist flexion task. We repeatedly (100 times) drew random samples of either narrow or wide spiking neurons, trained a decoding model, and then tested its performance on a separate set of data. We found that narrow spiking neural ensembles outperformed wide spiking neural ensembles across all muscles.

To further link narrow spiking neural activity with motor output, we examined data from an isometric center-out wrist task. Here, we attempted to predict the activity of 11 different upper limb muscles based on narrow and wide spiking neural ensembles (figure 8). We found that each muscle's activity was also better predicted by narrow spiking ensembles (median improvement in *R*^2^ was 0.06 across all motor features; ANOVA, EDC *F*_1,692_ = 525; APL *F*_1,692_ = 416; FDP *F*_1,692_ = 213; ECR *F*_1,692_ = 810; EDC2 *F*_1,692_ = 453; Brad *F*_1,692_ = 354; PT *F*_1,692_ = 488; FCU *F*_1,692_ = 219; FDS *F*_1,692_ = 335; FCR *F*_1,692_ = 90; FDS2 *F*_1,692_ = 625; *p* < 1e-8 for all muscles).

## Discussion

### Interpretation of narrow and wide spiking neural ensembles

It is tempting to assume that narrow and wide spiking neurons correspond to inhibitory interneurons and pyramidal cells, respectively, because generally, inhibitory interneurons exhibit narrow spike waveform widths while pyramidal cells have wider widths [37–40]. However, recent evidence suggests that such a clear delineation is unlikely. One study found a relationship between Betz cells, projection cells in layer V of MI, and spike waveform width, such that the largest Betz cells had the narrowest waveform widths [22]. This population of cells is thought to comprise approximately 10%–20% of neurons in layer V [41]; however, due to their large size, they are oversampled, and may actually represent closer to 50% of recorded projection neurons [41, 42]. Though the extent to which our data are subject to this sampling bias remains unknown, it is nevertheless likely that at least some of the narrow spiking neurons we recorded were indeed large projection neurons. Moreover, inhibitory interneurons exhibit a variety of spike widths including a small proportion with wide waveforms [23]. Thus, spike waveform width is not a reliable indicator of cell type.

In the present study, we found that narrow spiking neural ensembles substantially outperformed wide spiking ensembles in a variety of decoding contexts, and that this improvement in decoding performance was related to motor output. A fairly straightforward, albeit speculative explanation of this finding is that a substantial proportion of cells that we classified as narrow spiking neurons correspond to the largest Betz cells, and thus, the activity of the narrow spiking neural population contains more direct information about efferent motor activity.

Using Gaussian mixture models, we found that the distribution of spike waveform widths was best described by a mixture of two Gaussians based on AIC values; however, this finding does not imply that the true distribution of waveform widths is bimodal, nor are we arguing that it is. Indeed, the spike waveform width distribution was more clearly bimodal in some datasets than others. We used the Gaussian mixture model as a principled way of identifying the boundary between narrow and wide populations. In this way, it represents an improvement over previous methods based on specifying an arbitrary threshold [21]. The mixture model also provided a quantitative means of assessing the modality of the waveform width distribution rather than assuming bimodality.

### Application to a clinically relevant BMI

Recent reports have shown that small ensembles of neurons are capable of achieving a high degree of decoding performance [3, 43]. As BMIs scale to increasingly large degrees of freedom, these small ensembles may be used to control individual DoFs. In one study, individual control dimensions were allocated 10 neurons based on a statistical selection criterion [44]. In our data, we observed that small ensembles of narrow spiking neurons could achieve performance comparable to, if not better than large ensembles of wide spiking neurons. This suggests that a dynamic allocation scheme could be devised based on the width of recorded neurons such that some DoFs would be controlled by small ensembles of narrow spiking neurons, while other DoFs would be controlled by larger populations of wide spiking neurons yet each DoF would have the same expected level of performance despite being controlled by a different number of neurons.

An additional hurdle impeding the development of a clinically viable BMI is that few properties of the neural response are stable over long periods of time [45]. Here, we found that the bimodal distribution of spike waveform widths was similar across a timespan of several months. Additionally, in every dataset we analyzed, we observed a variety of spike waveform widths. Although there was some variability in the boundary between narrow and wide populations across animals, we observed both narrow and wide populations of cells in seven datasets recorded from five animals.

In summary, our approach has been to identify physiological properties of neurons that may reveal their utility in a neural decoder; this approach is not incompatible with other techniques aimed at improving decoding performance. Indeed, other approaches including linear dimensionality reduction and statistical model selection could be used in conjunction with waveform information to identify neurons within the narrow spiking neural population that are most relevant for decoding. More generally, we emphasize that neural decoding algorithms may be improved by using the underlying biological properties of neural signals to inform the design of these algorithms.

## Figures and Tables

**Table 1:** Summary of datasets Details regarding task, All times are listed in microseconds.

Dataset	Task	# data points	# training points	# test points	# narrow	# neurons	*μ*_1_	*σ*_1_	*μ*_2_	*σ*_2_	*π*	*λ*
rs050225	center-out	4233	3175	1058	71	105	224	53	434	37	0.68	0
rs051013	center-out	4423	3317	1106	42	57	235	67	438	54	0.73	0
rj040114	center-out	2374	1781	593	98	163	200	38	344	62	0.62	0
rj040207	center-out	5095	3821	1274	88	130	201	38	367	57	0.65	0
b080725	RTP	4631	3473	1158	62	87	221	62	383	78	0.70	1
mk080828	RTP	6225	4668	1557	38	51	223	38	416	52	0.74	0
j141203	wrist	23 982	17 987	5995	58	75	253	76	401	109	0.67	1

Note: dataset size, number of neurons, and fit parameters for the Gaussian mixture model.
